# Social support services for dementia during the COVID‐19 pandemic: A longitudinal survey exploring service adaptations in the United Kingdom

**DOI:** 10.1111/hex.13784

**Published:** 2023-05-29

**Authors:** Thaïs Caprioli, Clarissa Giebel, Siobhan Reilly, Hilary Tetlow, Stan Limbert, Mari Lloyd‐Williams

**Affiliations:** ^1^ NIHR ARC NWC Liverpool UK; ^2^ Department of Primary Care and Mental Health University of Liverpool Liverpool UK; ^3^ Bradford Dementia Group University of Bradford Bradford UK

**Keywords:** COVID‐19 pandemic, dementia, postdiagnostic support, social care, social support services

## Abstract

**Objectives:**

To understand how the delivery of dementia‐related social support services across the UK adapted during the pandemic.

**Methods:**

We devised a two‐part online and telephone longitudinal survey. Providers participated between March and June 2021, and again 3 months later. Information relating to services delivered and delivery methods employed was collected before and during the pandemic at two timepoints (T1 and T2).

**Results:**

A total of 75 participants completed the survey at T1, with 58 participants completing the survey at both timepoints. Thirty‐six participants had complete data at T1. Day care centres and support groups were the most delivered primary services. During the pandemic, services shifted from in‐person to remote or hybrid. While in‐person services started to resume at T2, most services remained hybrid. At T2, the frequency of service delivery increased, however, a decreasing trend in usage was observed across survey timepoints. The telephone was the most employed format to deliver remote and hybrid services, however, reliance on videoconferencing software significantly increased at T1. Videoconferencing software was often used alongside the telephone and emails to remotely deliver services.

**Conclusions:**

Services were able to adapt and provide support to some service recipients. Complementing novel approaches to service delivery with more traditional formats may facilitate access to service recipients with limited digital literacy. Following the easing of public health measures, many service recipients may be reluctant to engage with in‐person services. Thus, the provision of in‐person and remote services needs to be carefully balanced amidst the current hybrid landscape.

**Patient or Public Contribution:**

Two public advisors (a former unpaid carer and a person living with dementia) were involved in designing and piloting the tool, interpreting the results and disseminating the findings. Both public advisors have experience in delivering dementia‐related social support services before and or during the pandemic in the United Kingdom.

## 1 INTRODUCTION

Dementia is a growing public health issue. Characterised by declining cognitive functioning, dementia holistically impacts individuals' daily life^1^, ^2^ There are currently over 920,000 people with dementia in the United Kingdom, a number which is projected to rise to over a million by 2024.^3^ In the absence of a cure, equal access to responsive postdiagnostic support that addresses the needs of people with dementia and their unpaid carers is important to living well with the condition.^4^

Postdiagnostic support includes all services that respond to the changing support and care needs throughout the disease trajectory.^5^ Good postdiagnostic support is characterised by the timely identification of needs and provision of holistic support.^6^ Social support services form part of postdiagnostic support, and can be defined as nonclinical services that aim to support community‐dwelling people with dementia and unpaid carers to live well and independently. In the United Kingdom, social support services are provided by a mixture of public, private and voluntary sectors^7^ and delivered across various care settings. Services may include peer support groups, befriending services, day care centres, social activities, respite care and paid home care.

The usage of social support services is likely to vary throughout the disease trajectory. Possible benefits include promoting independence and reducing feelings of loneliness among people with dementia.^8^, ^9^ Social support services may facilitate opportunities to engage in meaningful activities, which is considered important to living well with dementia.^10^, ^11^ Furthermore, unpaid carers may equally benefit from accessing social support services, and psychoeducation and psychosocial interventions are thought to positively impact on the perceived caregiving burden and depressive symptoms.^12^, ^13^ While the use of social support services should be guided by people with dementia and unpaid carers' changing needs and preferences, access remains unequal.^14^, ^15^, ^16^ Barriers to access are numerous, and may include rural residency and being from an ethnic minority or a socioeconomically deprived background.^17^, ^18^, ^19^

The coronavirus (COVID‐19) pandemic significantly impacted the lives of people with dementia and unpaid carers,^20^, ^21^ and is likely to have engendered long‐term consequences.^22^ Many social support organisations were required to stop in‐person service delivery for extensive periods. The diminished opportunities to engage in activities and to socialise with others is thought to have contributed to the overall worsening of dementia symptoms, and general wellbeing of people with dementia and unpaid carers.^23^, ^24^, ^25^, ^26^ Challenged with a lack of available respite care and often undertaking additional caregiving duties, some unpaid carers reported an increase in caregiving burden.^21^ Nevertheless, some social support organisations adopted novel approaches to service delivery and continued to provide support to people with dementia and unpaid carers.

The reliance on the use of information communications technologies (ICT) across the service delivery landscape increased during the pandemic. Research exploring the use of ICT to conceivably facilitate timely and flexible access to remote post‐diagnostic support has largely focused on the medical management of people with dementia^27^, ^28^ and or reducing the caregiving burden.^29^ However, before^30^, ^31^, ^32^ and during the pandemic,^33^, ^34^, ^35^, ^36^ some social support services for people with dementia or the dyad were delivered remotely. Largely delivered by videoconferencing software, services included peer support groups,^34^ memory cafes^36^ and exercise health^30^, ^32^, ^33^ and singing classes.^35^ Accessing support remotely appears feasible, nevertheless, difficulties while navigating ICT were often experienced. Furthermore, while remote services were generally well received, some people with dementia and unpaid carers preferred accessing services in person.^36^

Despite a growing evidence base outlining the impact of the COVID‐19 pandemic on people with dementia and unpaid carers,^20^, ^21^ little is known about how social support services across the United Kingdom have adapted during the pandemic from the service providers' perspective. Therefore, this longitudinal online and telephone survey sought to compare (1) the types of social support services delivered and (2) the delivery methods employed; before and at two time points during the pandemic. Findings may provide insights into planning the delivery of social support services in prospective public health crises and possibly help satisfy the nationwide digital transformation agenda for health and care services.^37^

## 2 MATERIALS AND METHODS

### 2.1 Participants and recruitment

This study defined social support providers as someone who delivers nonclinical community‐based social support services to people with dementia and/or to unpaid carers in a paid or unpaid (volunteer) capacity. Eligible social support providers met the following criteria: (1) currently working (paid capacity) or helping/volunteering (unpaid capacity) to deliver social support services within the public, private of voluntary sectors in the United Kingdom; (2) delivering social support services to people with dementia and/or to unpaid carers living in the community; (3) either directly involved in delivering social support services or involved in the management/organisation of the delivery of social support services and (4) at least 18 years of age.

Social support providers were recruited through convenience sampling. We contacted social support organisations (publicly available email addresses and telephone numbers), engaged with social prescribing routes, advertised the study on social media (Twitter) and in internal university newsletters. Social support providers who have participated in our study will henceforth be referred to as ‘participants’ and the term ‘service recipients’ refers to people with dementia or unpaid carers who have accessed social support services delivered by participants in our study.

### 2.2 Data collection

The study involved completing a survey at two time points (T1 and T2). T1 included retrospective details about prepandemic service delivery and current data. This generated three time points for analysis. The data collection period for the survey at T1 lasted from the 25th of March to 4th of June 2021. Participants were reinvited, via email, to complete the survey at T2 3 months after they had completed the survey at T1. Three weeks were allocated to complete the survey at T2, with, if required, reminders sent to participants every 6 days after the initial invitation to complete the survey at T2. Data collection for the survey at T2 took place between 25th of June and 22nd of September 2021. Participants could choose to complete surveys online (Jisc Online Surveys) or by telephone. Where these were undertaken by telephone, a research team member scheduled a call and entered a participant's answers on the online version of the survey on their behalf.

The government‐imposed public health restrictions differed slightly across the nation. At T1 the restrictions were commencing to ease, gradually allowing a limited number of people to meet outdoors and, subsequently, indoors. Most restrictions were removed or considerably less restrictive at T2. A summary of the government‐imposed public health measures across the United Kingdom at each survey timepoint can be found in Supporting information: File S1.

### 2.3 Variables and tool

Information on the following variables were collected:

1. Characteristics of participants (age range, gender, ethnicity, years of full‐time education completed and years of working or volunteering as a social support provider).
2. Characteristics of provider (sector, current contractual arrangement (paid/unpaid), postcode of service provision area).
3. Services delivered (types of services delivered, identification of primary service [primary service refers to the service participants were delivering the most]).
4. Delivery methods employed (in‐person, hybrid or remote, use of digital and non‐digital formats).
5. Usage of primary service (service recipients; number of service recipients accessing service during a regular month).

The first survey obtained information on all variables listed above, and elicited information on service delivery before the pandemic (retrospectively) and at T1. The second survey solely obtained information regarding service delivery at T2.

The survey was piloted with a public advisor and with other research team members before data collection. Both public advisors and one member of the research team have experience in delivering dementia‐related social support service before and or during the pandemic in the United Kingdom.

### 2.4 Data analysis

Data were exported from Jisc Online Surveys to Microsoft Excel. All analysis was conducted in SPSS (version 27). The sample characteristics, services delivered and delivery methods employed were summarised at each survey time point using descriptive statistics. The Shapiro–Wilko test was used to test for normality. The number of hours worked/volunteered prior to the pandemic and at T1 was compared using the Wilcoxon‐signed rank test and compared across the three survey timepoints using the Friedman test.

Collected postcodes enabled the index of multiple deprivation (IMD) quintile to be generated for each service provision area. Although the calculation of the IMD differs slightly across the countries within the United Kingdom, the index provides a relative measure of neighbourhood deprivation by considering multiple factors including income, employment, education and health. The IMD score is ranked into five quintiles, with the first representing the most deprived and the fifth the least deprived areas.^38^ Obtaining the IMD quintiles of service provision areas may help to discover discrepancies in adaptation of service delivery across the United Kingdom.

## 3 RESULTS

### 3.1 Survey completion

**Figure 1:**
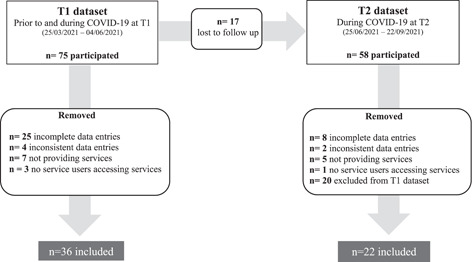
Flowchart depicting participation flow and completion rates during the longitudinal study. The uppermost boxes indicate the number of participants who completed the survey at each timepoint. Following the removal of (1) incomplete data entries (missing responses), (2) inconsistent data entries (contradicting responses within the same data entry), (3) participants not delivering social support services, (4) no service recipients accessing social support services (5) losses to follow up (participants who did not complete the survey at both time points), a total of 36 participants has complete data at T1 and 22 participants had complete data at both survey timepoints.

A total of 75 participants completed the survey at T1, and 58 participants completed the survey at both timepoints. Two participants at T1 and one at T2 completed the survey by telephone. The remaining participants completed the surveys online. As outlined in Figure 1, following the cleaning of the data sets, a total of 36 (48.0%) participants had complete data entries at T1, and 22 (37.9%) participants at all survey timepoints. Only participants with complete data entries were included in the analysis.

### 3.2 Participant and provider characteristics

Most participants identified as female, White and at least 35 years old. Participants mainly delivered services in a paid capacity, within the third sector and in areas classified within the third and fifth IMD quintiles (Table 1).

**Table 1:** Participant sociodemographic characteristics (T1: *n* = 36; T2: *n* = 22).

*n* (%)	T1 data set (*n* = 36)	T2 data set (*n* = 22)
Gender		
Female	27 (75.0)	18 (81.8)
Male	9 (25.0)	4 (18.2)
Age group		
25–34	1 (2.8)	1 (4.5)
35–44	9 (25.0)	8 (36.4)
45–54	5 (13.9)	3 (13.6)
55–64	13 (36.1)	6 (27.3)
65 And over	8 (22.2)	4 (18.2)
Ethnicity		
White	34 (94.4)	21 (95.5)
Mixed/multiple ethnic groups	2 (5.6)	1 (4.5)
Sector		
Public	3 (8.3)	3 (13.6)
Private	2 (5.6)	1 (4.5)
Third sector	31 (86.1)	18 (81.8)
Contractual arrangement		
Paid	24 (66.7)	17 (77.3)
Unpaid	12 (33.3)	5 (22.7)
Region		
East of England	6 (16.7)	5 (22.7)
Greater London	3 (8.3)	1 (4.5)
North East	1 (2.8)	1 (4.5)
North West	7 (19.4)	4 (18.2)
South East	3 (8.3)	–
South West	7 (19.4)	5 (22.7)
West Midlands	5 (13.9)	3 (13.6)
Yorkshire and Humber	3 (8.3)	2 (9.1)
Scotland	1 (2.8)	1 (4.5)
IMD quintile of service provision area in England^a^		
1	4 (11.1)	1 (4.5)
2	5 (13.9)	2 (9.1)
3	10 (27.8)	8 (36.4)
4	6 (16.7)	5 (22.7)
5	10 (27.8)	5 (22.7)
Years full‐time education		
Median (range)	16.5 (4–23)	17 (7–20)
Years working/volunteering as a social support provider^b^		
Median (range)	10.5 (1.5–42)	11.5 (1.5–39)

Abbreviation: IMD, index of multiple deprivation.

^a^ Excludes one participant delivering support services in Scotland. Area categorised within the third quintile within the Scottish classification.

^b^ Refers to the cumulative number of years working/volunteering to deliver social support services to any service recipients, not necessarily within dementia care.

While the numbers of hours worked/volunteered slightly reduced at T1, participants worked or volunteered similar hours across survey timepoints (median [range]: prior COVID‐19: 29 [2–50]; T1: 21.3 [1–60]; *Z* = −0.853, *p* = .394); (median [range]: prior‐COVID‐19: 30 [2–40]; T1: 25.5 [2–40]; T2: 31.5 [1.5–45]; *X* ^2^ = 0.304, *p* = .859). A total of nine participants (25.0%) paused service delivery at the beginning of the pandemic (median: 6 weeks, range: 2–45 weeks).

### 3.3 Service provision before and during the pandemic

Participants delivered a median of two services across all survey timepoints. Social activities and support groups, followed by day care centres and accompanying/befriending were the most delivered services (Supporting Information: Table S2).

#### 3.3.1 Types of primary services delivered

A total of 28 participants (77.8%) delivered the same primary service before the pandemic and at T1. Thirteen participants (59.1%) delivered the same primary service across all survey timepoints. Support groups, day care centres and social activities were the most delivered primary services across survey timepoints. Services were mainly delivered to people with dementia and unpaid carers (Tables 2 and 3).

**Table 2:** A summary of primary services delivered, service recipients and usage per delivery method before the pandemic and at T1 (*n* = 36).

	Prior COVID‐19, *n* = 36 (*n* [%])				T1 *n* = 36 (*n* [%])			
	In person (*n* = 26)	Hybrid (*n* = 9)	Remotely (*n* = 1)	Total (*n* = 36)	In person (*n* = 4)	Hybrid (*n* = 17)	Remotely (*n* = 15)	Total (*n* = 36)
Primary services delivered								
Accompanying/befriending	1 (3.8)	1 (11.1)	–	2 (5.6)	1 (25.0)	2 (11.8)	1 (6.7)	4 (11.1)
Advice, support and information	2 (7.7)	3 (33.3)	1 (100.0)	6 (16.7)	–	3 (17.6)	2 (13.3)	5 (13.9)
Day care centre	9 (34.6)	1 (11.1)	–	10 (27.8)	3 (75.0)	6 (35.3)	–	9 (25.0)
Social activities	5 (19.2)	2 (22.2)	–	7 (19.4)	–	3 (17.6)	5 (33.3)	8 (22.2)
Support groups	9 (34.6)	2 (22.2)	–	11 (30.6)	–	3 (17.6)	7 (46.7)	10 (27.8)
Service recipients of primary services								
Unpaid carer (current/former)	1 (3.8)	–	–	1 (2.8)	–	–	2 (13.3)	2 (5.6)
Person with dementia	7 (26.9)	–	–	7 (19.4)	1 (25.0)	2 (11.8)	–	3 (8.3)
Both	18 (69.2)	9 (100.0)	1 (100.0)	28 (77.8)	3 (75.0)	15 (88.2)	13 (86.7)	31 (86.1)
How often primary services were delivered remotely^a^								
Never	24 (92.3)	–	–	24 (66.7)	3 (75.0)	–	–	3 (8.3)
Sometimes	2 (7.7)	7 (77.8)	–	9 (25.0)	1 (25.0)	7 (41.2)	2 (13.3)	10 (27.8)
Often	–	1 (11.1)	–	1 (2.8)	–	7 (41.2)	1 (6.7)	8 (22.2)
All of the time	–	1 (11.1)	1 (100.0)	2 (5.6)	–	3 (17.6)	12 (80)	15 (41.7)
Satisfaction with primary service delivery								
Very satisfied	21 (80.8)	6 (66.7)	1 (100.0)	28 (77.8)	–	4 (23.5)	6 (40.0)	10 (27.8)
Fairly satisfied	4 (15.4)	3 (33.3)	–	7 (19.4)	2 (50.0)	13 (76.5)	8 (53.3)	23 (63.9)
Not satisfied	1 (3.8)	–	–	1 (2.8)	2 (50.0)	–	1 (6.7)	3 (8.3)
Average number of times primary services were delivered^a^								
Median (range)	8.5 (1–40)	4 (1–29)	24	7.5 (1–40)	20 (5–24)	5 (1–25)	6 (1.5–30)	7 (1–30)
Average number of service recipients accessed primary services^a^								
Median (range)	27.5 (5–180)	40 (12–320)	100	30 (5–320)	10 (5–21)	25 (3–150)	30 (4–220)	24 (3–220)

^a^ During a regular month.

**Table 3:** A summary of primary services delivered, service recipients and usage per delivery method at T1 and T2 (*n* = 22).

	T1 *n* = 22 (*n* [%])				T2 *n* = 22 (*n* [%])			
	In person (*n* = 2)	Hybrid (*n* = 12)	Remotely (*n* = 8)	Total (*n* = 22)	In person (*n* = 4)	Hybrid (*n* = 14)	Remotely (*n* = 4)	Total (*n* = 22)
Primary services delivered								
Accompanying/befriending	1 (50.0)	–	–	1 (4.5)	–	1 (7.1)	–	1 (4.5)
Advice, support and information	–	2 (16.7)	1 (12.5)	3 (13.6)	–	2 (14.3)	–	2 (9.1)
Day care centre	1 (50.0)	5 (41.7)	–	6 (27.3)	2 (50.0)	5 (35.7)	–	7 (31.8)
Social activities	–	2 (16.7)	4 (50.0)	6 (27.3)	–	2 (14.3)	4 (100.0)	6 (27.3)
Support groups	–	3 (25.0)	3 (37.5)	6 (27.3)	2 (50.0)	4 (28.6)	–	6 (27.3)
Service recipients of primary services								
Unpaid carer (current/former)	–	–	1 (12.5)	1 (4.5)	–	–	1 (25.0)	1 (4.5)
Person with dementia	–	2 (16.7)	–	2 (9.1)	2 (50.0)	2 (14.3)	–	4 (18.2)
Both	2 (100.0)	10 (83.3)	7 (87.5)	19 (86.4)	2 (50.0)	12 (85.7)	3 (75.0)	17 (77.3)
How often primary services were delivered remotely^a^								
Never	1 (50.0)	–	–	1 (4.5)	3 (75.0)	1 (7.1)	–	4 (18.2)
Sometimes	1 (50.0)	4 (33.3)	1 (12.5)	6 (27.3)	1 (25.0)	5 (35.7)	–	6 (27.3)
Often	–	6 (50.0)	–	6 (27.3)	–	7 (50.0)	–	7 (31.8)
All of the time	–	2 (16.7)	7 (87.5)	9 (40.9)	–	1 (7.1)	4 (100.0)	5 (22.7)
Satisfaction with primary service delivery								
Very satisfied	–	3 (25.0)	3 (37.5)	6 (27.3)	4 (100.0)	4 (28.6)	1 (25.0)	9 (40.9)
Fairly satisfied	1 (50.0)	9 (75.0)	5 (62.5)	15 (68.2)	–	10 (71.4)	3 (75.0)	13 (59.1)
Not satisfied	1 (50.0)	–	–	1 (4.5)	–	–	–	–
Average number of times primary services were delivered^a^								
Median (range)	12.5 (5–20)	10 (1–20)	8 (2–22)	8 (1–22)	12.5 (4–46)	14 (2.5–32)	3 (1–50)	12 (1–50)
Average number of service recipients accessed primary services^a^								
Median (range)	14.5 (5–24)	27.5 (15–100)	40 (4–220)	27.5 (4–220)	23 (6–46)	28.3 (10–104)	23.5 (5–358)	26.8 (5–358)

^a^ During a regular month.

#### 3.3.2 Delivery methods and formats employed to deliver primary services

A shift from in‐person to hybrid or remote services was observed at T1 (Table 2). At T2, the number of remote services halved, and most services were hybrid (Table 3).

Telephone calls, emails, website resources and the post were used to deliver remote and hybrid services before the pandemic. While the telephone remained the most employed format to deliver remote and hybrid services during the pandemic, the use of videoconferencing software significantly increased at T1 (Tables 4 and 5). Compared to prior to the pandemic, 22 more participants used videoconferencing software at T1, and the format was most employed to deliver remote or hybrid support groups, advice and information provision and social activities (Table 6). Thirteen participants used videoconferencing software to remotely deliver services at T1 (Table 4). Apart from three participants (23.1%) who solely used videoconferencing software, most participants complemented the use of videoconferencing software with the telephone (*n* = 9 [69.2%]) and or emails (*n* = 7 [53.8%]). The two participants (13.3%) who did not use videoconferencing software, remotely delivered services by formats including telephone and post (Table 4).

**Table 4:** A summary of formats employed to deliver hybrid and remote primary services before the pandemic (*n* = 10) and at T1 (*n* = 32) (overall data set *n* = 36).

	Prior COVID‐19 *n* = 10 (*n* [%])			T1 *n* = 32 (*n* [%])		
	Hybrid (*n* = 9)	Remote (*n* = 1)	Total (*n* = 10)	Hybrid (*n* = 17)	Remote (*n* = 15)	Total (*n* = 32)
Formats employed to deliver primary service remotely^a^						
e‐learning	–	–	–	2 (11.8)	–	2 (6.3)
e‐mail	8 (88.9)	1 (100.)	9 (90.0)	11 (64.7)	8 (53.3)	19 (59.4)
Internet forum	–	–	–	1 (5.9)	–	1 (3.1)
Internet portal	–	–	–	2 (11.8)	1 (6.7)	3 (9.4)
Mobile applications	–	–	–	–	1 (6.7)	1 (3.1)
Post	6 (66.7)	1 (100.0)	7 (70.0)	6 (35.3)	6 (40.0)	12 (37.5)
Social media	4 (44.4)	1 (100.0)	5 (50.0)	6 (35.3)	3 (20.0)	9 (28.1)
Telephone calls	9 (100.0)	1 (100.0)	10 (100.0)	16 (94.1)	11 (73.3)	27 (84.4)
Text messaging	4 (44.4)	–	4 (40.0)	8 (47.1)	4 (26.7)	12 (37.5)
Videoconferencing	3 (33.3)	–	3 (30.0)	12 (70.6)	13 (86.7)	25 (78.1)
Website resources	7 (77.8)	1 (100.0)	8 (80.0)	5 (29.4)	6 (40.0)	11 (34.4)
Average number of formats employed per participant						
Median (range)	5 (2–7)	5 (0–0)	5 (2–7)	3 (1–7)	4 (1–7)	3.5 (1–7)

^a^ Participants could select multiple formats.

**Table 5:** A summary of formats employed to deliver hybrid and remote primary services at T1 (*n* = 20) and at T2 (*n* = 18) (overall data set *n* = 22).

	T1 *n* = 20 (*n* [%])			T2 *n* = 18 (*n* [%])		
	Hybrid (*n* = 12)	Remote (*n* = 8)	Total (*n* = 20)	Hybrid (*n* = 14)	Remote (*n* = 4)	Total (*n* = 18)
Formats employed to deliver primary service remotely^a^						
e‐learning	1 (8.3)	–	1 (5.0)	–	–	–
e‐mail	7 (58.3)	3 (37.5)	10 (50.0)	9 (64.3)	–	9 (50.0)
Internet forum	1 (8.3)	–	1 (5.0)	1 (7.1)	–	1 (5.6)
Internet portal	2 (16.7)	1 (12.5)	3 (15.0)	2 (14.3)	–	2 (11.1)
Post	3 (25.0)	3 (37.5)	6 (30.0)	7 (50.0)	–	7 (38.9)
Social media	5 (41.7)	2 (25.0)	7 (35.0)	5 (35.7)	1 (25.0)	6 (33.3)
Telephone calls	11 (91.7)	7 (87.5)	18 (90.0)	12 (85.7)	3 (75.0)	15 (83.3)
Text messaging	5 (41.7)	1 (12.5)	6 (30.0)	6 (42.9)	–	6 (33.3)
Videoconferencing	10 (83.3)	6 (75.0)	16 (80.0)	9 (64.3)	3 (75.0)	12 (66.7)
Website resources	3 (25.0)	4 (50.0)	7 (35.0)	5 (35.7)	1 (25.0)	6 (33.3)
Average number of formats employed per participant						
Median (range)	3 (1–7)	3.5 (1–5)	3 (1–7)	4 (1–9)	1.5 (1–3)	3.5 (1–9)

^a^ Participants could select multiple formats.

**Table 6:** Formats employed per hybrid or remote primary service at T1 (*n* = 32) (overall data set *n* = 36).

	Accompanying/befriending (*n* = 3)	Advice, information and support (*n* = 5)	Day care centre (*n* = 6)	Social activities (*n* = 8)	Support groups (*n* = 10)	Total (*n* = 32)
Formats employed to deliver primary services remotely^a^						
e‐learning	1 (33.3)	–	1 (16.7)	–	–	2 (6.3)
e‐mail	2 (66.7)	3 (60.0)	4 (66.7)	7 (87.5)	3 (30.0)	19 (59.4)
Internet forum	–	–	1 (16.7)	–	–	1 (3.1)
Internet portal	–	1 (20.0)	2 (33.3)	–	–	3 (9.4)
Mobile application	–	–	–	–	1 (10.0)	1 (3.1)
Post	2 (66.7)	4 (80.0)	2 (33.3)	3 (37.5)	1 (10.0)	12 (37.5)
Social media	–	1 (20.0)	4 (66.7)	2 (25.0)	2 (20.0)	9 (28.1)
Telephone calls	3 (100.0)	5 (100.0)	6 (100.0)	7 (87.5)	6 (60.0)	27 (84.4)
Text messaging	2 (66.7)	2 (40.0)	4 (66.7)	1 (12.5)	3 (30.0)	12 (37.5)
Videoconferencing	1 (33.3)	4 (80.0)	4 (66.7)	6 (75.0)	10 (100.0)	25 (78.1)
Website resources	2 (66.7)	2 (40.0)	2 (33.3)	4 (50.0)	1 (10.0)	11 (34.4)
Average number of formats employed per participant						
Median (range)	6 (1–6)	5 (2–7)	5.5 (3–7)	3.5 (2–7)	2 (1–7)	3.5 (1–7)

^a^ Participants could select multiple formats.

At T1 and T2, a small number of participants employed formats to deliver remote and or hybrid services (e‐learning, internet forums and internet portals) that were not used before the pandemic (Tables 4 and 5).

#### 3.3.3 Frequency of service delivery and usage of primary services

**Figure 2:**
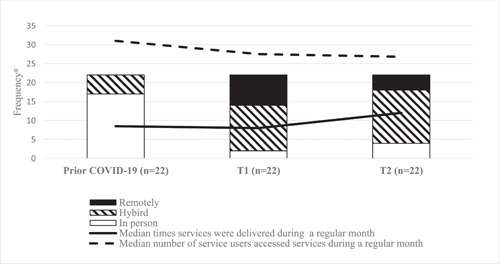
Graph showing the shift in service delivery method, frequency of service delivery and usage of primary services across the three timepoints (*n* = 22). ^α^Frequency relates to: number of services/average number of service recipients accessed services per regular month/average number of times services were delivered per regular month.

Across the survey timepoints, the frequency of service delivery per regular month increased, and a small, but important, decreasing trend in usage was observed (Figure 2 and Supporting Information: Table S2). Compared to before the pandemic, in‐person services at T1 were delivered more frequently but accessed by almost a third of service recipients (Table 2). At T2, the use of in‐person services increased and the use of hybrid services remained similar to T1 (Table 3).

#### 3.3.4 COVID‐19 mitigation measures implemented to deliver primary services

All participants implemented mitigation measures when delivering in‐person services (either as part of fully in‐person or hybrid delivery models) at T1 and T2. Social distancing, wearing personal protective equipment and the COVID‐19 vaccination of social support providers, were the most implemented measures (Table 7).

**Table 7:** A summary of COVID‐19 mitigation measures employed to deliver in‐person or hybrid primary services at T1 (*n* = 14) and T2 (*n* = 18) (overall data set *n* = 22).

	T1 *n* = 14 (*n* [%])	T2 *n* = 18 (*n* [%])
COVID‐19 measures employed to deliver primary services^a^		
Service recipients vaccinated	10 (71.4)	12 (66.7)
Service recipients bring food/drink	3 (21.4)	3 (16.7)
Service recipients contact details (track and trace)	5 (35.7)	8 (44.4)
Different activities	11 (78.6)	10 (55.6)
Minimise contact with service recipients	10 (71.4)	9 (50.0)
Shorter time spent with service recipients	5 (35.7)	3 (16.7)
Social distancing	14 (100.0)	16 (88.9)
Social support providers vaccinated	12 (85.7)	13 (72.2)
Wearing PPE^β^	12 (85.7)	12 (66.7)
Average number of measures employed per participant		
Median (range)	6 (2–9)	5 (2–9)

Abbreviation: PPE, personal protective equipment.

^a^ Participants could select multiple measures.

#### 3.3.5 Satisfaction with primary service delivery

Before the pandemic, participants were most satisfied when delivering services in‐person (Table 2). At T1 and T2, remote and hybrid services yield similar satisfaction levels, and most providers were ‘fairly satisfied’ (Table 3).

## 4 DISCUSSION

This exploratory longitudinal study is one of the first to report on how dementia‐related social support services have adapted during the pandemic. While a slight decrease at T1, participants worked or volunteered a comparable number of hours across the survey timepoints. Day centres, social activities and support groups were the most delivered primary services. During the pandemic, a shift from in‐person to remote or hybrid services was observed. Although in‐person services started to resume at T2, services were largely delivered by hybrid approaches. While the frequency of service delivery increased at T2, a small, but important, decreasing trend in usage was observed across survey timepoints. The reduced access aligns with previous research outlining the alarming consequences following the reduced service use during the pandemic.^23^, ^24^ This study adds to the growing evidence base by exploring the issue from the service delivery angle.

The adaptation of delivery methods is likely to depend on various factors, which may differ across sectors and or by organisation size. Factors may include social support providers' access to ICT equipment, digital literacy and ability to receive support to navigate the technology.^35^, ^39^, ^40^, ^41^ While the telephone was the most employed format, akin to other studies, we observed an increased reliance on the use of videoconferencing software during the pandemic.^33^, ^34^, ^35^, ^36^ The use of videoconferencing software peaked when public health restrictions were most restrictive, and may have been used as an interim to see one another when doing so in person was unsafe. Delivering services by videoconferencing software is likely to have engendered a steep learning curve, which may include adapting the content delivered^35^, ^40^, ^41^ and identifying how to optimally facilitate engagement with people with dementia and unpaid carers.^33^, ^34^ Delivering services by videoconferencing software may be time effective^27^ and, compared to the telephone, the format offers visual cues which may help foster a sense of connection with people with dementia and unpaid carers.^33^ However, compared to delivering services in person, the use of videoconference software may present some drawbacks. For example, some providers reported challenges in assessing and progressing people with dementia participating in an exercise health programme,^33^ a lack of privacy during some medical follow‐ups^27^ and the audio latency formed a barrier to engaging in singing groups.^35^ While not collected in our survey, the use of videoconferencing software to deliver support asynchronously, largely by prerecorded videos, is documented in other studies.^35^, ^41^, ^42^ This format may help to overcome issues relating to audio latency and facilitate timely and flexible access to support. Further research is required to explore the challenges and opportunities presented by using different ICT formats to deliver an array of services across the three sectors.

The ability to access, use and benefit from services delivered by videoconferencing software is unlikely to be equal among people with dementia and unpaid carers. Factors that may facilitate engagement may include higher socioeconomic status,^43^ less severe dementia stage and the ability to receive support to navigate and engage with remote services.^33^, ^44^ Most participants in our survey employed more than one format to remotely deliver services, and the use of videoconferencing software was frequently coupled with telephone calls and emails. During the pandemic, the option to access remote services by means other than videoconference has been reported elsewhere,^35^, ^42^, ^45^ and is likely to facilitate access to people with limited digital literacy. Concerningly, we observed a reduced service usage during the pandemic. However, the option to access services by more traditional formats, alongside novel approaches, is likely to have helped to mitigate the widening of inequalities in access. Although not collected in our survey, other studies have sought to bridge people with dementia and unpaid carers' limited ICT access and literacy by providing devices and ongoing support throughout the study period.^31^, ^32^ The planning of future service delivery may benefit from gaining an understanding of whether similar approaches have been employed during the pandemic, and whether these have facilitated access to services. However, being able to access support remotely may not necessarily translate to usage. People with dementia and unpaid carers have reported that accessing services remotely is ‘not the same’ as in‐person services,^34^, ^36^ and their preferences on how support is accessed needs to be considered.

Although not a new delivery method in itself, we observed that reliance on hybrid services significantly increased during the pandemic. In our survey, hybrid services continued to feature as public health measures eased, and subsequently, removed. People with dementia and unpaid carers who accessed remote services during the pandemic valued the support received, however, some preferred in‐person services.^36^ Once legally allowed, services gradually began to deliver socially distanced in‐person services, often in small groups and or outdoors.^39^, ^42^ This aligns with our findings, and is likely to explain the increased frequency but fewer service recipients accessing in‐person services at T1. Nevertheless, the opportunity to access services remotely following the removal of the public health measures is likely to facilitate access to some service recipients in our survey. Subsequent to 2 years of confinement, people with dementia and unpaid carers may remain cautious and fear engaging in‐person activities.^22^, ^39^ In addition, many people experienced a deterioration in their dementia symptoms,^20^, ^22^ and accessing services in person may no longer be feasible or desirable. Thus, the ongoing opportunity to access services remotely, while in‐person services are resuming, is likely to be important for some people with dementia and unpaid carers. Across the survey timepoints, we observed a slight change in the formats employed to deliver hybrid services. While the telephone and email remained frequently used throughout, the use of videoconferencing peaked when public health was most restrictive. Further research is required to characterise the ongoing delivery of hybrid services which may help to guide the transition from emergency to routine service delivery following future public health crises.

## 5 LIMITATIONS

This appears to be the first survey to date which captured the fast‐moving changes in adapting and delivering dementia‐related social support services for dementia during the pandemic, from the service provider point of view. Given its novelty and attempt to longitudinally report on a broad range of social support services, this exploratory survey has multiple strengths. However, some limitations ought to be acknowledged while interpreting our findings. While our sample demographics are akin to those of the adult social care sector,^46^ our sample lacked ethnic diversity and largely compromised of participants working or volunteering within the third sector in England and Scotland. Therefore, our study provides a much required first snapshot of a variety of dementia‐related social support services, with data collected at a time when service providers were particularly struggling for time to engage with anything other than to provide support to people with dementia and unpaid carers. Despite piloting the tool before data collection, we identified that the wording of some questions could have been clarified. We noticed some discrepancies in how the same participants answered related questions, rendering it difficult, at times, to understand which formats were employed to deliver social support services remotely, and those used to engage with service provide support to service recipients outside of attending services. This may have been avoided had the tool been piloted amongst providers delivering different services across various sectors. The collected satisfaction levels provide a general insight into participants' satisfaction with primary service delivery methods throughout survey timepoints and would benefit from further (possibly qualitative) exploration. Lastly, our tool only identified formats used to deliver services. Including a follow‐up question asking participants to quantify how often they employed each format(s) to deliver primary services at each survey timepoint would have strengthened our tool.

## 6 CONCLUSIONS

During the pandemic, we observed a shift from in‐person to remote or hybrid services. Compared to before the pandemic, following the removal of public health measures, services were delivered more frequently, but accessed by fewer service recipients. While the telephone remained the most employed format, reliance on videoconferencing software significantly increased during the pandemic. Employing videoconferencing software alongside more traditional formats, such as telephone calls and emails, is likely to facilitate access to service recipients with limited digital literacy. Nevertheless, the ongoing opportunity to access services remotely while in‐person services resume is likely to facilitate access to some service recipients. Thus, it is important that the provision of in‐person and remotely delivered services is carefully balanced amidst the current hybrid landscape.

## AUTHOR CONTRIBUTIONS

*Conception, study design, execution, acquisition of data, analysis, interpretation of results, drafting or writing of the manuscript*: Thaïs Caprioli. *Conception, study design, supervision of Thaïs Caprioli, interpretation of results, editing and reviewing the manuscript*: Clarissa Giebel, Siobhan Reilly and Mari Lloyd‐Williams. *Study design, interpretation of results, editing and reviewing the manuscript*: Hilary Tetlow. *Interpretation of results, editing and reviewing the manuscript*: Stan Limbert.

## ACKNOWLEDGEMENTS

The authors would like to thank all the participants who have taken the time to participate in our study.

## CONFLICT OF INTEREST STATEMENT

The authors declare no conflict of interest.

## ETHICS STATEMENT

Ethics approval was obtained from the University of Liverpool before commencing the study (Ref: 9817). The study information sheet was disseminated to all participants and online written informed consent was obtained before participation.

## Supporting information

Supplementary table 1 A summary of key governmental imposed public health measures at T1 and T2. (Summaries obtained from England: Institute for Government analysis, 2021; Department of Health and Social Care, 2021.Scotland: Scottish Parliament Information Centre, 2022. Wales: Welsh Parliament, 2021; 2022. Northern Ireland: The executive Office, 2021a ‐e.). Supplementary table 2: An overall longitudinal summary of primary services delivered, service recipients and usage prior to the pandemic, T1 and T2 (n = 22). ^α^ Participants could select multiple services ^β^ During a regular month.

## Data Availability

The data that support the findings of this study are available from the corresponding author upon reasonable request.
