# Supplementary material for: Social support services for dementia during the COVID‐19 pandemic: A longitudinal survey exploring service adaptations in the United Kingdom
Source: Health Expect. 2023 May 29;26(4):1726–37. doi: 10.1111/hex.13784 (PMC10349216; doi:10.1111/hex.13784)
Supplement: Supplementary file 1 — Supplementary table 1 A summary of key governmental imposed public health measures at T1 and T2. (Summaries obtained from England: Institute for Government analysis, 2021; Department of Health and Social Care, 2021.Scotland: Scottish Parliament Information Centre, 2022. Wales: Welsh Parliament, 2021; 2022. Northern Ireland: The executive Office, 2021a ‐e.). Supplementary table 2: An overall longitudinal summary of primary services delivered, service recipients and usage prior to the pandemic, T1 and T2 (n = 22). α Participants could select multiple services β During a regular month. [file HEX-26--s001.docx]

**Supplementary Table 1.** A summary of key governmental imposed public health measures at T1 and T2. (Summaries obtained from England: Institute for Government analysis, 2021; Department of Health and Social Care, 2021. Scotland: Scottish Parliament Information Centre, 2022. Wales: Welsh Parliament, 2021; 2022. Northern Ireland: The executive Office, 2021a ‐e.).

| **Survey timepoint** | **Months**  **2021** | **Implemented COVID-19 public health measures** | | | |
| --- | --- | --- | --- | --- | --- |
|  |  | **England** | **Scotland** | **Wales** | **Northern Ireland** |
| T1 | March | **29^th^ March**: Outdoor gatherings of either six people or two households allowed. Removal of stay at home order. |  | **25^th^ March:** Stay local restrictions removed. |  |
|  | April | **12^th^ April:** Non-essential retail, hairdressers and public buildings reopen. Outdoor venues reopen. No indoor mixing between different households allowed. | **2^nd^ April:** Local non-essential journeys allowed.  **5^th^ April**: Hairdressers, garden centres re-open.  **16^th^ April**: Travel within Scotland for outdoor socialising, recreation and exercise allowed. Outdoor meetings of groups of up to six adults from up to six households. | **12^th^ April: N**on-essential retail opens. Travel from Wales to UK allowed.  **24^th^ April:** Six people allowed to meet outdoors.  **26^th^ April:** re-opening of outdoor hospitality and indoor entertainment. Organised outdoor activities for up to 30 people | **23^rd^ April**: Close-contact services and and outdoor attractions re-open.  **30^th^ of April**: Non-essential retail and outdoor hospitality reopen. Up to 15 people from three households allowed to gather in outdoor domestic settings. |
|  | May | **17^th^ May:** A maximum of 30 people allowed to mix outdoors. ‘Rule of six’ or two households allowed to mix indoors. Indoor venues reopen. | **17^th^ May:** Eased restrictions on hospitality, entertainment and education. In level 2 areas, indoor gatherings of six people from three households allowed. | **3^rd^ May:** Reopening of gyms and leisure centres.  **17^th^ May**: Six people from six households meet indoors. Re-opening of indoor hospitality and indoor entertainment. Increase number of people who can attend organised indoor / outdoor activities. | **24^th^ May:** Indoor hospitality reopens. Six people from two households can mix indoors in domestic settings. Up to 15 people from three households can meet outdoors in a domestic garden. Face coverings mandatory in indoor public settings. |
|  | June |  |  | **7^th^ June:** Up to 30 people can meet outdoors. Up to three household can form an extended household. Increase number of people allowed to attend organised outdoor gatherings.  **17^th^ June:** Four weeks pause in easing of restrictions due to new delta variant. |  |
| T2 | July | **19^th^ July:** Most legal limits on social contact removed. Final closed sectors of the economy re-open. | **19^th^ July:** Scotland moves to level 0. Physical distancing reduced to one meter indoor / outdoor places. Outdoor gatherings of up to 15 people from five households allowed without physical distancing. Face coverings remain. | **17^th^ July:** Up to six people meet indoors. Increase in the number of people who can attend indoor events. Limit of people attending outdoor events removed. Face coverings remain mandatory in most indoor public places. | **27^th^ of July:** Up to ten people from three households will be allowed to meet indoors in a domestic setting. |
|  | August | **16^th^ August:** Fully vaccinated adults no longer required to self-isolate following contact with COVID-19 case | **9^th^ August:** Legal requirement for physical distancing and limits on gatherings removed. Two-meter physical distancing remains in place in health and care settings. Use of face coverings indoors remains. Removal of self-isolation rules for fully vaccinated people who test negative to COVID-19. Work from home advice remains. | **7^th^ August:** Removal of the limit of people meeting indoors. All businesses re-open. Face coverings no longer required in most hospitality nut required in health and care settings and public transport. Fully vaccinated people only need to self-isolate if they develop symptoms or test positive to COVID-19. Work from home advice remains. | **16^th^ August:** People who are fully vaccinated will no longer require to self-isolate for 10 days following contact with a positive COVID-19 case. The limit of people meeting in domestic outdoor settings was removed. |
|  | September |  |  |  | **10^th^ September:** Indoor gatherings in domestic settings of 15 people from four households allowed. Removal of table service in indoor / outdoor places. |

**Supplementary Table 2.** An overall longitudinal summary of primary services delivered, service recipients and usage prior to the pandemic, T1 and T2 (n = 22). ^α^Participants could select multiple services ^β^During a regular month.

|  |  | **Prior COVID-19 (n=22) (n (%))** | **T1 (n=22) (n (%))** | **T2 (n=22) (n (%))** |
| --- | --- | --- | --- | --- |
| **All services delivered** ^α^ | | | | |
|  | Accompanying/ befriending | 7 (31.8) | 8 (36.4) | 6 (27.3) |
|  | Advice, support and information | 3 (13.6) | 5 (22.7) | 6 (27.3) |
|  | Day care centre | 8 (36.4) | 7 (31.8) | 7 (31.8) |
|  | Home meal delivery | - | 1 (4.5) | - |
|  | Paid home care | 1 (4.5) | 2 (9.1) | 1 (4.5) |
|  | Respite care | 3 (13.6) | 1 (4.5) | 1 (4.5) |
|  | Social activities | 16 (72.7) | 15 (68.2) | 13 (59.1) |
|  | Support groups | 17 (77.3) | 12 (54.5) | 15 (68.2) |
|  | Transport | 2 (9.1) | 1 (4.5) | 3 (13.6) |
| **Average number of services delivered per participant** | | | | |
|  | Median (range) | 2.5 (1-5) | 2 (1-6) | 2 (1-7) |
| **Primary services delivered** | | | | |
|  | Accompanying/ befriending | 1 (4.5) | 1 (4.5) | 1 (4.5) |
|  | Advice, support and information | 3 (13.6) | 3 (13.6) | 2 (9.1) |
|  | Day care centre | 7 (31.8) | 6 (27.3) | 7 (31.8) |
|  | Social activities | 3 (13.6) | 6 (27.3) | 6 (27.3) |
|  | Support groups | 8 (36.4) | 6 (27.3) | 6 (27.3) |
| **Service recipients of primary services** | | | | |
|  | Unpaid carer (current / former) | 1 (4.5) | 1 (4.5) | 1 (4.5) |
|  | Person with dementia | 5 (22.7) | 2 (9.1) | 4 (18.2) |
|  | Both | 16 (72.7) | 19 (86.4) | 17 (77.3) |
| **Primary services delivery models** | | | | |
|  | In person | 17 (77.3) | 2 (9.1) | 4 (18.2) |
|  | Hybrid | 5 (22.7) | 12 (54.5) | 14 (63.6) |
|  | Remotely | - | 8 (36.4) | 4 (18.2) |
| **How often primary services were delivered remotely** ^β^ | | | | |
|  | Never | 15 (68.2) | 1 (4.5) | 4 (18.2) |
|  | Sometimes | 6 (27.3) | 6 (27.3) | 6 (27.3) |
|  | Often | 1 (4.5) | 6 (27.3) | 7 (31.8) |
|  | All of the time | - | 9 (40.9) | 5 (22.7) |
| **Overall satisfaction with primary service delivery** | | | | |
|  | Very satisfied | 16 (72.7) | 6 (27.3) | 9 (40.9) |
|  | Fairly satisfied | 5 (22.7) | 15 (68.2) | 13 (59.1) |
|  | Not satisfied | 1 (4.5) | 1 (4.5) | - |
| **Average number of times primary services were delivered** ^β^ | | | | |
|  | Median (range) | 8.5 (1-40) | 8.0 (1-22) | 12 (1-50) |
| **Average number of service recipients accessed primary services** ^β^ | | | | |
|  | Median (range) | 31 (5-180) | 27.5 (4-220) | 26.8 (5-358) |

References

1. Department of Health and Social Care. Self‐isolation removed for double‐jabbed close contacts from 16 August. 2021. <https://www.gov.uk/government/news/self-isolation-removed-for-double-jabbed-close-contacts-from-16-august>
2. Institute for Government analysis. Timeline of UK government coronavirus lockdowns and measures, March 2020 to December 2021. 2021. <https://www.instituteforgovernment.org.uk/sites/default/files/chart-images/timeline-coronavirus-lockdown-december-2021_0.png>
3. Scottish Parliament Information Centre (SPICe). Timeline of coronavirus (COVID‐19) in Scotland. 2022. <https://spice-spotlight.scot/2022/12/16/timeline-of-coronavirus-covid-19-in-scotland/>
4. The Executive Office. Executive agrees relaxations to Covid restrictions. 2021. <https://www.executiveoffice-ni.gov.uk/news/executive-agrees-relaxations-covid-restrictions>
5. The Exectutive Ofiice. Executive confirms relaxations to restrictions. 2021. <https://www.northernireland.gov.uk/news/executive-confirms-relaxations-restrictions>
6. The Executive Office. Executive decisions on relaxations to Covid‐19 restrictions. July 26, 2021. <https://www.executiveoffice-ni.gov.uk/news/executive-decisions-relaxations-covid-19-restrictions-26-july-2021>
7. The Executive Office. Statement on Executive decisions. August 12, 2021. <https://www.northernireland.gov.uk/news/statement-executive-decisions-12-august-2021>
8. The Executive Office. Statement on executive decisions. September 6, 2021. <https://www.executiveoffice-ni.gov.uk/news/statement-executive-decisions-6-september-2021>
9. Welsh Parliament. Coronavirus teimline: Welsh and UK government's response. 2021. <https://research.senedd.wales/media/po3nf424/coronavirus-timeline-5th-senedd-14-05-21.pdf>
10. Welsh Parliament.Coronavirus teimline: the response in Wales. 2022. <https://research.senedd.wales/media/e3aiug4j/coronavirus-timeline-26-09-22.pdf>
